# Perceptions of nurses on access to structural empowerment in a hospital in the Western Cape

**DOI:** 10.4102/curationis.v43i1.2018

**Published:** 2020-07-15

**Authors:** Gladness Roji, Karien Jooste

**Affiliations:** 1 Department of Nursing Science, Faculty of Health and Wellness Sciences, Cape Peninsula University of Technology, Cape Town, South Africa

**Keywords:** Structural empowerment, support, information, resources, opportunities

## Abstract

**Background:**

Structural empowerment is an ever-evolving concept interpreted and applied in many different ways as it focuses on the structures in a healthcare organisation to allow competent nurses to manage empowering opportunities in a professional manner. At a public hospital in the Western Cape, nurses complained about a lack of access to structural empowerment in a hospital, including structures of power, such as clear information, to partake in important decisions.

**Objectives:**

The purpose of this study was to describe how nurse managers could support nurses in accessing structural empowerment through power resources.

**Method:**

A quantitative design was followed with a survey. The accessible population in this study was different categories of nurses of professional, enrolled and assistant nurses (*N* = 200), which were on duty at the time of data gathering. The sample was selected by means of probability sampling (*n* = 110). An existing instrument based on a five-point Likert scale was distributed that took 45 minutes to complete. Descriptive and inferential statistics were calculated, and the chi-square was used to indicate statistical significance differences among the nursing categories on the items (*p* < 0.05).

**Results:**

The general results indicated that the majority of nurses had challenges to access structural empowerment through power sources (information, support and resources). Significant differences were found between nurse categories for having the necessary supplies for the job (*p* = 0.043) and rewards for unusual job performance (*p* = 0.023). Those aspects on which no significant differences were found are of utmost importance, as they indicate the urgency of addressing limitations in power sources for all categories of nurses.

**Conclusion:**

Empowerment can be achieved by enabling access to structural empowerment through power sources (i.e. opportunities, information, resources and support) at different levels for all categories of nurses.

## Introduction

Employee empowerment has always been an interesting domain for researchers as it supports organisations in enhancing the work performance of nurses and improves their work satisfaction (Raza et al. 2015:1). Empowerment is a management practice that is fundamental for professional growth of nurses and positively affects the quality of patient care (García-Sierra & Fernández-Castro 2018:2809). On the other hand, structural empowerment focuses on structures of, for example, power sources that enhance shared decision-making, influence of nurses, lifelong professional development, and strong community partnerships. This ensures that the organisation’s mission, vision and values come to life (Lippincott Solutions 2017).

The aim of empowerment is to create a healthcare organisation composed of skilled, competent nurses who fulfil their duties professionally while enjoying it, providing the best knowledgeable resources related to their performance, as required by the organisation (Saremi 2016:6). However, for work challenges, it could be expected that nurses have available structural empowerment in their work situation, either in the public or private healthcare sector organisations.

Laschinger et al. (2014:348) focus on *structural empowerment* as the extent to which work environments provide access to support, resources, information and opportunities to develop nurses’ skills. The availability of structural empowerment in an organisation is a managerial element that can improve the empowerment of nurses. Dubey and Singhal (2016:110) define structural empowerment as a mechanism that links and coordinates individuals within the framework of their roles, authority and *power sources*. Organisations should focus on structural empowerment that enhances relations, communication, decision-making processes, procedures and systems that assist employees with their responsibilities to reach the goals of an organisation. It also increases nursing autonomy, promoting the highest levels of clinical excellence and professional practice (Nurse.com 2019).

Structural empowerment entails transferring authority and power to lower categories of staff (Ashena & Keikha 2015:257). Harada and Mndoor (2016:2431) emphasise that structural empowerment focuses on the vital role of managers who provide information, rewards, knowledge and power to nurses, as well as guidance in the manner in which it should be implemented. Managers share power with nurses through specifying certain plans and objectives of the organisation and providing information and resources needed for nurses to deliver quality patient care (Salajeghe, Rezaei & Ahmadi 2015:94).

Wilson et al. (2015:12) state that structural empowerment could increase professional development and research opportunities and empower nurses to practise at their full potential. In this article power refers to having information, support and resources in the workplace of nurses.

## Research problem

During clinical nurse meetings in a public hospital in the Western Cape, the researcher heard various complaints about the lack of structural *empowerment* to enable nurses to address the challenging work situations they encounter. During these meetings, it was mentioned that nurses lacked power resources such as clear *information, resources and support* to partake in important decisions, while social connections with colleagues were challenging. Al-Dweik et al. (2015:3) also found that nurse managers failed to establish boundaries for employee empowerment to use power resources, as they mistrusted them with work-related decision-making. The overall research question that emerged was: *To what extent do nurses access structural empowerment through power resources in a public hospital in the Western Cape?*

## Purpose of the study

The purpose of this study was to describe how nurse managers could support nurses in accessing structural empowerment through power resources.

The objectives of the study were to:

- Describe nurses’ perceptions of access to structural empowerment through power resources in a public hospital.
- Describe how nurse managers could support nurses through power resources in accessing structural empowerment.

## Research design

The researcher used a descriptive, quantitative design to describe nurses’ perceptions of access to structural empowerment through power sources in the organisation. This approach assisted the researcher to describe data through statistics for analysis (Moore 2016:2).

### Population and sampling

At the time of the study, the accessible population in this study was all categories of nurses (*N* = 200) from a public hospital in the Western Cape. The sample formula of Krejcie and Morgan (1970:610) was used to determine the sample size for all nurse categories (at least 167 nurses of all categories). The accessible population served as the sample. Of the 110 nurses who completed the questionnaire, 40 were professional nurses (PNs), 31 were enrolled nurses (ENs) and 39 were enrolled nursing auxiliaries (ENAs).

### Data gathering

A survey was conducted with an existing instrument, the Conditions of Work Effectiveness Questionnaire (CWEQ-I), developed by Chandler in 1986. This instrument was based on Kanter’s theory of structural empowerment (1977). The CWEQ-I was pre-tested at a meeting with 13 nurses in a similar hospital, who were not part of the sample. Respondents were given an information sheet and a written consent form. Respondents completed the questionnaires, which took around 45 minutes to complete, in their own time over three months. Completed questionnaires were placed in envelopes and collected by the researcher. The CWEQ-I is a 58-item questionnaire with six subscales (structural empowerment elements) (Laschinger 2013:2). Items were rated on a five-point Likert-type response scale with scores ranging from 1 (none), very little, some, often, to 5 (a lot) on:

- Structures of opportunity (7 items).
- Power sources (24 items).
- Structures of social connections that include (27 items).

In this article the responses on the items of power sources are discussed.

### Data analysis

The Statistical Package for Social Sciences (SPSS), version 21.0 for Windows was used to analyse data. The descriptive statistics analysed measures of central tendency (mean), measures of spread (standard deviation) and measures of shape (skewness) of the nurses’ perceptions on the items. Data were presented in mathematical visuals of tables. Inferential statistical analysis was conducted as chi-square (χ^2^) was used to determine the significant differences between the mean values on power sources of structural empowerment for the three categories of nurses.

### Validity and reliability

This researcher used a classic questionnaire that has been used in numerous studies (Chandler 1986). The pre-test ensured that respondents understood the items stated in the questionnaire. Face validity involved an independent academic expert in research and management who looked at the items in the questionnaire and agreed that the items are a valid measure of the concept (Sangoseni, Hellman & Hill 2013:3). Cronbach’s alpha coefficient (α) was determined to assess the internal consistency of the questionnaire (Table 1). The results were compared with those Laschinger (1999) obtained. The results indicated moderate to high reliability. The mean structural empowerment score of all the items was 3.8. All three sections of the questionnaire on power sources obtained a Cronbach’s alpha (α) above the acceptable standard value of 0.7: access to information 0.92, access to support 0.92, and access to resources 0.91. The total Cronbach’s alpha coefficient for internal consistency was 0.91.

**TABLE 1:** Cronbach’s alpha coefficient (α). Conditions of Work Effectiveness Questionnaire.

Power sources of three sections (subscales) of structural empowerment in the instrument	Current study (2017)	Interpretation for current study	Laschinger (1999)
Information	0.92	Good	0.86
Support	0.92	Good	0.88
Resources	0.91	Good	0.81

### Ethical considerations

Ethical approval to conduct the study was granted by the Cape Peninsula University of Technology (CPUT) Ethics Committee (Ethical clearance number: CPUT/HW-REC 2013/H3720-09-13). The researcher requested permission from the relevant author to use the CWEQ-I questionnaire. Respondents were protected by not including any names or personal information to questionnaires to ensure anonymity and were informed that they had the right to decline and withdraw from the study at any time.

## Findings

A total of 110 (100%) nurses participated in the study, of whom 40 (36.6%) were PNs, 31 (28.1%) ENs and 39 (35.45%) ENAs. The majority of nurses who participated in the study were female (*n* = 100, 90.9%), while only 10 (9.1%) male nurses participated. The majority (72.6%) of respondents were 50 years and younger, while half of the respondents had between 10 and 20 years of work experience. The *items on power sources* related to the access to information, access to support and access to resources. The number of responses differed on the items.

### Access to information

No significant differences between nurse categories were found on the items related to their access to information as a source of power. The mean values of the responses of the categories ENs and ENAs were mostly higher on the items than the PNs. The importance of the findings of no significant differences implied that the majority of the three nurse categories had *very little* to *some* access to information. The items discussed show the aspects in which power sources are needed that are of great concern (Table 2). Responses of all categories of nurses indicated that they overall had only *some* information about the current state of the hospital, showing a skewness of 0.018 on responses.

**TABLE 2:** Items on access to information as a source of power for structural empowerment.

Items	Nurse categories	*n*	*x*	SD	χ^2^	*p*	Skewness
Being informed about the current state of the hospital (*n* = 106)	PNs	38	2.9	1.2	3.0	0.275	0.018
			3.1	-			
			3.0	-			
	ENs	29	3.1	1.3			
	ENAs	39	3.0	1.2			
Being informed about the relationship of nurses’ units to the rest of hospital (*n* = 106)	PNs	38	3.3	1.3	8.9	0.950	−0.395
	ENs	30	3.6	1.3			
	ENAs	38	3.3	1.3			
Being informed about how peers perform their duties (*n* = 104)	PNs	38	3.2	1.2	3.8	0.847	−0.265
	ENs	29	3.4	1.2			
	ENAs	37	2.9	1.4			
Being informed about values of top management (*n* = 108)	PNs	39	3.0	1.3	6.2	0.614	0.005
	ENs	30	3.1	1.4			
	ENAs	39	3.1	1.2			
Being informed about the goals of top management (*n* = 109)	PNs	39	2.7	1.2	10.0	0.259	0.007
	ENs	30	3.0	1.4			
	ENAs	38	3.0	1.3			
Being informed about the unit’s year plan (*n* = 109)	PNs	40	2.7	1.2	7.9	0.437	−0.028
	ENs	31	2.9	1.3			
	ENAs	38	2.9	1.4			
Being informed about how salary decisions are made for peers (*n* = 109)	PNs	39	2.0	1.1	14.9	0.060	0.359
	ENs	31	2.1	1.4			
	ENAs	39	2.7	1.2			
Being informed about what other departments think of your unit (*n* = 101)	PNs	36	2.7	1.3	7.0	0.533	−0.130
	ENs	29	3.3	1.1			
	ENAs	36	2.9	1.4			

PNs, professional nurses; ENs, enrolled nurses; ENAs, enrolled nursing auxiliaries.

The mean value of responses was 2.99, with a broad distribution of responses (SD = 1.215) around the mean value on access to information. The differences between responses of the three categories of nurses were not significant (χ^2^ = 3.0, *p* = 0.275) on having information around the hospital status.

Responses of PNs, ENs and ENAs indicated that they had inadequate (*sometimes* to *often)* information on how units fit into the broader picture of the hospital (the relationships between nurses’ units with the rest of the hospital), indicating a skewness of −0.395 and a mean value of the responses around working relationships of 3.36. The findings indicated that respondents differed in their opinions (SD = 1.274), although no significant difference (χ^2^ = 8.9, *p* = 0.395) between the responses of the three nurse categories was obtained. Nurses indicated a more positive response on having *some* information about other departments’ activities (skewness of −0.130) than the rest of the hospital. The overall mean value of the responses, on having knowledge of what other departments think of their units, was 2.95 with a broad distribution of responses (SD = 1.299), indicating a reasonably normal curve and no significant difference (χ^2^ = 7.0, *p* = 0.533) between the nurse categories.

A strategic work plan and job description for nurses seemed to be lacking. On the one hand, the responses of nurses on being informed about the unit’s year plan showed a skewness of −0.028. Nurses had *little* access to information, with a broad distribution of responses (SD = 1.278) indicating having various similar views on the year plan of all three categories of nurses (χ^2^ = 7.9, *p* = 0.437). On the other hand, more respondents indicated they *often* performed their duties in relation to how peers in similar positions did their work (skewness of −0.265). The mean value of the responses was 3.15, with a broad distribution of responses (SD = 1.283). The different categories of nurses did not significantly differ (χ^2^ = 3.8, *p* = 0.847) in the rating of the items. Nurses needed access to information on the values and goals of top management in their institution. Firstly, all nurses had *some* access to information regarding the values of top management, showing a skewness of 0.005 of responses. Respondents had a broad and normal distribution of responses (SD = 1.249) indicating that the three categories of nurses did not differ on the need to know more about the values of top management (χ^2^ = 6.2, *p* = 0.614).

Secondly, respondents had *some* information about the goals of top management and the responses showed a skewness of 0.007. The absence of clear goals to guide the service was confirmed by the low mean value of 2.91, with respondents having various reasons (a broad distribution of responses) on the lack of information on the goals set for the institution in which they rendered a service (SD = 1.263). The three categories of nurses did not have a significant difference (χ^2^ = 10.0, *p* = 0.259) on their perceptions of lacking goals to work towards.

Respondents indicated *some* to *often* involvement in how salary decisions for nurses in similar positions were made, showing a positive skewness of 0.359 (Table 2). All categories of nurses showed similar responses (χ^2^ = 14.9, *p* = 0.060).

### Access to support

Respondents had *some* access to specific information on good performance and responses showed a skewness of −0.222 (Table 3). The overall mean value of the responses was 3.22, with a broad distribution of responses (SD = 1.329), indicating a reasonably normal curve. The three categories of nurses did not have a significant difference (χ^2^ = 7.0, *p* = 0.528) in their responses.

**TABLE 3:** Items on access to support as a source of power for structural empowerment.

Items	Nurse categories	*n*	*x*	SD	*χ*^2^	*p*	Skewness
Specific information on good performance (*n* = 109)	PNs	40	2.9	1.2	7.0	0.528	−0.222
	ENs	30	3.5	1.4			
	ENAs	39	3.4	1.3			
Specific comments on performance that could improve (*n* = 108)	PNs	40	3.1	1.3	11.7	0.165	−0.310
	ENs	30	3.2	1.2			
	ENAs	38	3.5	1.4			
Helpful hints or problem-solving advice (*n* = 106)	PNs	39	2.9	1.2	11.3	0.183	−0.282
	ENs	28	3.0	1.3			
	ENAs	39	3.5	1.3			
Information or suggestions about job possibilities (*n* = 106)	PNs	40	2.7	1.2	10.0	0.260	−0.045
	ENs	29	3.0	1.3			
	ENAs	37	3.2	1.4			
Involvement in discussions on further training and education (*n* = 106)	PNs	39	2.5	1.3	3.5	0.895	0.241
	ENs	29	2.7	1.4			
	ENAs	38	2.8	1.5			
Access to help when there is a work crisis (*n* = 106)	PNs	39	2.6	1.3	12.6	0.124	−0.69
	ENs	28	3.4	1.5			
	ENAs	39	3.4	1.4			
Access to people who can get the job done (*n* = 108)	PNs	40	2.8	1.2	7.3	0.498	−0.066
	ENs	29	3.5	1.3			
	ENAs	39	3.2	1.3			
Help in getting material and supplies needed (*n* = 106)	PNs	38	2.8	1.1	10.6	0.223	−0.012
	ENs	29	3.3	1.5			
	ENAs	39	3.1	1.5			
Rewards and recognition for a job well done (*n* = 107)	PNs	39	2.1	1.3	6.8	0.551	0.431
	ENs	29	2.7	1.6			
	ENAs	39	2.8	1.6			

PNs, professional nurses; ENs, enrolled nurses; ENAs, enrolled nursing auxiliaries.

The findings indicated that performance appraisal of nurses, through feedback on their work performances and receiving rewards and recognitions for tasks well done, needed intervention by top management.

A negative skewness of −0.310 was found in relation to the responses to nurses receiving specific comments on their work performance that could improve (Table 3). The overall mean value of 3.23 indicated that respondents only received *some* information on the improvement of their performance, which could indicate the lack of knowing on which key areas to improve. The broad distribution of responses (SD = 1.287), indicated all categories having different viewpoints on the lack of performance feedback of similar nature (χ^2^ = 11.7, *p* = 0.165). On the other hand, more negative responses were found on rewards for a job well done. A lower mean value of 2.53 with a broader distribution of responses (SD = 1.5) were found. A positively skewness of 0.431 was indicated in relation to receiving rewards and recognition for a job well done.

Advice to solve problems in the workplace, assistance in a work crisis and support in obtaining equipment seemed to be limited. All the nurse categories indicated receiving only *some* helpful hints or problem-solving advice, showing a negative skewness of −0.282. The overall mean value of the responses was 3.13 (SD = 1.265), with no significant difference between the nurse categories (χ^2^ = 11.3, *p* = 0.183). The different categories of nurses all had a need for helpful hints in the workplace that could enhance their performance. Problem-solving advice, to help in a work crisis, is also essential. Responses relating to the availability of help in a work crisis showed a negative skewness of −0.069 and an overall mean value of 3.1. Respondents only indicated *some* assistance in a work crisis, with a lack of problem-solving and crisis management (SD = 1.447; χ^2^ = 12.6, *p* = 0.124). Similarly, the three nurse categories showed only having *some* support when accessing people who can get the job done (= 3.12; SD = 1.28). The findings showed a skewness of −0.066 and no difference between the responses of the categories (χ^2^ = 7.3, *p* = 0.498). Support related to getting help when obtaining the necessary materials and supplies showed a negative skewness of −0.012 with no significant difference (χ^2^ = 10.6, *p* = 0.223) between the responses of the three nurse categories. The overall mean value of the responses was 3.6, with a broad distribution of responses around the mean value (SD = 1.365), indicating a normal curve.

Staff development and promotion opportunities were a great concern. The three nurse categories indicated similar responses in only having *some* information or suggestions about job possibilities (χ^2^ = 10.0, *p* = 0.260). The lack of knowledge about possible job opportunities showed a reasonably normal curve (SD = 1.265) indicating various opinions on the lack of information on their future career ladder.

Respondents indicated *little* involvement in the discussion of further training and education, showing a skewness of 0.241. The overall mean value of the responses was 2.68, with a broad distribution of responses around the mean value (SD = 1.418), indicating diverse viewpoints on staff development opportunities in the workplace. Similar responses (χ^2^ = 3.5, *p* = 0.895) between the responses of the three nurse categories on involvement in discussions on training were obtained.

### Access to resources

The mean values of the responses of the categories were mostly similar on all the items. The majority of the three nurse categories had *very little* to *some* access to resources. Respondents *sometimes* had the necessary supplies to use in the completion of their duties (Table 4). Responses showed a negative skewness of −0.096. The overall mean value of the 108 (100%) responses was 2.97, with a broad distribution of responses (SD = 1.148), indicating a normal curve of different opinions. A significant difference (χ^2^ = 15.9, *p* = 0.043) was obtained between the responses of the three different categories of nurses in respect of their access to the necessary supplies.

**TABLE 4:** Items on access to resources as a source of power for structural empowerment.

Items	Nurse categories	*n*	*x*	SD	χ^2^	*p*	Skewness
Access to supplies necessary for the job (*n* = 108)	PNs	40	3.1	0.9	15.9	0.043	−0.096
	ENs	29	3.1	1.4			
	ENAs	39	2.7	1.2			
Time available for necessary paperwork (*n* = 107)	PNs	40	2.8	1.1	5.3	0.715	0.101
	ENs	28	2.9	1.3			
	ENAs	39	2.7	1.2			
Time available for job requirements (*n* = 108)	PNs	40	2.8	1.1	4.3	0.822	−0.131
	ENs	29	2.9	1.3			
	ENAs	39	2.7	1.2			
Acquiring temporary help when needed (*n* = 108)	PNs	40	2.5	1.1	9.4	0.308	0.231
	ENs	29	2.9	1.3			
	ENAs	39	3.0	1.3			
Influencing decisions to obtain human resources (*n* = 106)	PNs	40	2.6	1.1	8.3	0.403	0.011
	ENs	27	2.9	1.2			
	ENAs	39	2.8	1.2			
Influencing decisions to obtain unit supplies (*n* = 105)	PNs	39	2.7	1.1	6.4	0.596	0.040
	ENs	27	2.7	1.2			
	ENAs	39	2.7	1.2			
Influencing decisions to obtain equipment for units (*n* = 104)	PNs	38	2.8	1.1	15.4	0.050	0.001
	ENs	27	2.7	1.2			
	ENAs	39	2.7	1.3			

PNs, professional nurses; ENs, enrolled nurses; ENAs, enrolled nursing auxiliaries.

The findings indicated concerns around time management and support. Responses related to the availability of time to do paperwork showed an overall mean value of 2.81 for the 107 (100%) respondents. A low mean value of 2.81, with a broad distribution of responses around the mean value (SD = 1.199), indicated that respondents lacked time for paperwork (Table 4). Similarly, for the item on time available to accomplish job requirements, the overall mean value of responses was 2.95, indicating disagreement with meeting job expectations in a reasonable timeframe. The broad distribution of responses (SD = 1.155) indicates that some respondents of all groups agreed more than others on being able to do their job within the time (χ^2^ = 4.3, *p* = 0.822). The three nurse categories indicated they received *little* temporary help when needed during accomplishment of their job requirements. The overall mean value of 2.79, with a wide distribution of responses (SD = 1.223) was obtained between the categories on temporary help when needed.

Respondents needed resources to make decisions. Responses relating to their influence on decisions to obtain human resources for their units showed a skewness of 0.011 (SD = 1.161). The low overall mean value of 106 (100%) respondents was 2.75, showing the need for having influence as leaders to take a decision to get hold of staff when needed. A lower overall mean value of 2.71 showed a lack among different categories of nurses of the power to influence decisions to obtain supplies for their unit. The broad distribution of responses (SD = 1.15) indicates a positively skewed curve confirming the need to make decisions. Similarly, all the responses relating to influencing decisions to obtain equipment for units showed an overall low mean value of 2.76 with a positive skewness of −0.001 (SD = 1.17). There was a significant difference (χ^2^ = 15.4, *p* = 0.050) between the responses of the three categories indicating that different categories had different influential positions.

## Discussion

Structural empowerment through power sources related to (1) access to information, (2) access to support and (3) access to resources.

The low mean values obtained on the items regarding information sharing indicated that all categories of nurses needed more information in the workplace to fulfil their job requirements. In general, PNs knew less than ENs about the current state of the hospital, which is disturbing as PNs are the leaders of lower categories. If leaders know less than their followers do, patient care outcomes will be negatively influenced and their authority undermined. Elnaga and Imran (2014:13) state that fully empowered employees should have all the necessary information about the state of an organisation.

Nurses as providers of healthcare cannot work in isolation, but it seemed there was a lack of a strategic plan that could enhance the relationship nurses have while working between units and their workplace in the broader hospital context, which is necessary to strengthen service delivery and a shared vision. Vorina, Simonič and Vlasova (2017:246) state that employee involvement is the goal of strategic initiatives and an essential element of organisational health, designed to improve employees’ attitudes towards leaders and co-workers.

All three nurse categories indicated they had *little* to *some* access to information from managers on the values and goals of top management. Organisational values are needed as they inform employees about management’s expectations of their performance on all levels (Glavas 2016:796). The findings indicated a lack of information given by managers to nurses on the year plan of the unit. Unit planning as a continuous process begins with the PNs setting goals, which should result in a plan of action to accomplish them (Lawrence 2015).

The majority of respondents lacked information from their managers on decision-making, such as how salary decisions were made. A lack of understanding around decisions made could play a role in lack of collaboration. However, nurses in the public sector will not truly know how salary decisions are made, because the South African government makes those decisions after collaboration with the bargaining chamber and trade unions (Ditlopo et al. 2013:140). A previous study using the CWEQ-I instrument indicated that using power sources is one of the characteristics of the decision-making process, together with the use of a unique body of knowledge (Teixeira & Figueredo 2015).

Access to support should be through feedback, guidance, emotional support, helpful advice or hands-on assistance, as well as problem-solving advice, which can all be very beneficial to nurses (Hagerman et al. 2017:2). Some respondents in this study did receive support through problem-solving advice on improving performance; however, problem-solving advice could include emotional support, helpful advice or hands-on assistance in becoming empowered (Bish, Kenny & Nay 2012:3). Respondents received some suggestions about their job possibilities, but perceived an absence of discussions about further training and education, and career management.

Professional nurses usually assist followers in a work crisis, but the responses indicated that only some support was given with limited rewards for performances. Structural empowerment through rewards and recognition for a job well done, for example, could contribute to work satisfaction (Yürümezoğl & Kocaman 2019). A previous study that used the CWEQ-I instrument found structural empowerment useful in designing organisational strategies, for which empowering employees may be advantageous to improve the quality of services as well as increasing employees’ well-being (Sadeghi-Gandomani, Alavi & Afshar 2019).

Respondents indicated that they were *rarely* allowed to acquire the access to resources, such as the temporary help needed for their units. The role of PNs in managerial positions is to obtain human resources as a leader, but the results indicated that ENs and ENAs acted in these positions, which could show a lack of human resources. Professional nurses and not their subordinates should pay attention to strategies that address the immediate work environment, and the progression of nursing careers (Vari et al. 2016:1047). According to the results, ENs had the most time available to fulfil job requirements. However, PNs are those who need time to assist lower categories of nurses in daily planning and more advanced tasks such as coordinating care resulting from the doctors’ ward rounds, and ensuring that the treatment is given or that X-rays and blood tests, for example, are done (Armstrong, Rispel & Penn-Kekana 2015:4).

The CWEQ-I instrument was used in a study that found that structural empowerment among nurses contributes to the assessment of tendencies of nurses to behave autonomously in the various clinical settings, and that structural empowerment is an essential prerequisite for nursing practice (Sadeghi-Gandomani et al. 2019). Nurses *sometimes* could influence decisions to obtain human resources and supplies for their units. This study confirmed the findings of Heydari, Najar and Bakhshi (2015:391), that professional nurses in a higher position have the most influence on obtaining equipment as they are responsible for the correct use and repairs of equipment through teaching followers how to use it. In a study on work effectiveness using the CWEQ-I instrument, creating conditions that allow for individual development and provide access to decision-making processes is essential (Orłowska & Łaguna 2018).

## Recommendations

Nurses should have **access to information** related to the current status of a healthcare institution (Walston 2012:11).

- Managers should inform staff members about the organisational policies, protocols and circulars. Yasothai, Jauhar and Bashawir (2015:94) emphasise that employees need information to deal with new challenges and complete expected tasks adequately.
- Reward systems should be known to staff, and be aligned to the organisation’s goals, objectives, strategy, team performance and staff development. Management can use the reward strategy to attract qualified staff and, in so doing, maintain a highly motivated workforce in an organisation.
- Nurse management needs to communicate the roles and responsibilities of each nurse category to eliminate any role confusions between nurse categories. Different means of communication could enhance communication that should be freely used and available with a focus on technology and mobile devices. Use of emails ensures a fast spread of information from top management, while meetings ensure that information reaches the majority of staff accurately (Skytt et al. 2015:1008).

Mostefalder and Al-Sulaiti (2015:2) refer to **access to support** as receiving feedback and guidance from seniors and peers.

- Nurse managers should encourage staff to work independently, while supporting them with decision-making and teamwork in their daily challenges. For the latter, they should equip themselves professionally with knowledge and experience to act as a role model. The nurse manager should act as a role model for nurse categories to understand the complexities of nurses’ work, and to advocate in the best interests of nurses and patients.
- Nurse management must coach nurses and clarify the nature and content of delegated tasks (Naujokaitiene, Tereseviciene & Zydziunaite 2015:4). A safe environment allows nurses to ask questions and to rely on senior co-workers and supervisors for assistance and advice (Read & Laschinger 2015:3).
- Nurses should have **access to adequate resources**: supplies, materials, money and sufficient time to perform duties. Nurse managers should have adequate staff within budgetary constraints in order for nurses to provide adequate patient care, with material resources available to perform their essential duties. Nurses with a wide range of skills and knowledge must be able to access equipment and material resources in order to maximise their potential. Sufficient human resources within the organisation are essential, as their availability at all times contributes to the accomplishment of assigned tasks. Employees must furthermore be able to access temporary help when needed (Wanjiku, Gachungu & Kabere 2016:126).

The results of this study could lay the foundation for further studies on nurses’ perceptions of the availability of organisational power structures at a public hospital, with the emphasis on perceptions of PNs as shift leaders in the absence of the unit manager. The results may also provide nursing leadership with a useful starting point for evaluating the accessibility of current structural empowerment, as well as their roles in the availability thereof for PNs.

## Limitations of the study

The study was conducted in only one public hospital in the Western Cape and cross-sectional, total sampling was used. Therefore, the findings cannot be generalised more broadly to other hospitals. Time was a factor that may have negatively affected the filling in of the questionnaire as nurses only had a shift to complete the questionnaires.

## Conclusion

Nurses’ perceptions of their access to organisational structures at a public hospital in the Western Cape varied in this quantitative study, but the overall majority of all categories of nurses indicated having minimal access to empowerment power structures. It could be assumed that good structural conditions within workplaces are essential to nurses’ well-being, and their ability to access structural empowerment is largely dependent on management. Nurses need a workplace that promotes empowerment, managers who believe in their abilities and acknowledgement that there is power in work relationships and in the care nurses provide.
